# A new *Vibrio cholerae* sRNA modulates colonization and affects release of outer membrane vesicles

**DOI:** 10.1111/j.1365-2958.2008.06392.x

**Published:** 2008-08-15

**Authors:** Tianyan Song, Franziska Mika, Barbro Lindmark, Zhi Liu, Stefan Schild, Anne Bishop, Jun Zhu, Andrew Camilli, Jörgen Johansson, Jörg Vogel, Sun Nyunt Wai

**Affiliations:** 1 Department of Molecular Biology, Umeå UniversitySE-901 87 Umeå, Sweden; 2 RNA Biology Group, Max Planck Institute for Infection Biology10117 Berlin, Germany; 3 Departments of Microbiology, Physics, and Biology, University of PennsylvaniaPhiladelphia, PA 19104, USA; 4 Howard Hughes Medical Institute and the Department of Molecular Biology an Microbiology, Tufts University School of MedicineBoston, MA 02111, USA

## Abstract

We discovered a new small non-coding RNA (sRNA) gene, *vrrA* of *Vibrio cholerae* O1 strain A1552. A *vrrA* mutant overproduces OmpA porin, and we demonstrate that the 140 nt VrrA RNA represses *ompA* translation by base-pairing with the 5′ region of the mRNA. The RNA chaperone Hfq is not stringently required for VrrA action, but expression of the *vrrA* gene requires the membrane stress sigma factor, σ^E^, suggesting that VrrA acts on *ompA* in response to periplasmic protein folding stress. We also observed that OmpA levels inversely correlated with the number of outer membrane vesicles (OMVs), and that VrrA increased OMV production comparable to loss of OmpA. VrrA is the first sRNA known to control OMV formation. Moreover, a *vrrA* mutant showed a fivefold increased ability to colonize the intestines of infant mice as compared with the wild type. There was increased expression of the main colonization factor of *V. cholerae*, the toxin co-regulated pili, in the *vrrA* mutant as monitored by immunoblot detection of the TcpA protein. VrrA overproduction caused a distinct reduction in the TcpA protein level. Our findings suggest that VrrA contributes to bacterial fitness in certain stressful environments, and modulates infection of the host intestinal tract.

## Introduction

*Vibrio cholerae* is a Gram-negative bacterium that causes the acute, severe diarrhoeal disease cholera. Its natural ecosystem includes aquatic environments in endemic locations. Two factors are critical to *V. cholerae* virulence – cholera toxin (CT) and an intestinal colonization factor known as the toxin co-regulated pilus (TCP). Poorly characterized environmental cues influence the expression of CT and TCP *in vivo* (Faruque *et al*., 1998). Two sensory proteins, ToxR and TcpP, likely play a role in detection of the environmental signals, and activate the transcription of genes involved in TCP and CT expression through the expression of ToxT (Lee *et al*., 1999).

Outer membrane vesicles (OMVs) are produced by a wide variety of Gram-negative bacteria (Beveridge, 1999) including *Vibrio* species (Kondo *et al*., 1993) during their growth. They contain outer membrane proteins, lipopolysaccharides, phospholipids and, as the vesicles are being released from the surface, they entrap some of the underlying periplasm. Different hypotheses have been proposed for the function of OMVs. OMVs have been suggested to promote the adherence, the transfer of bacterial DNA and the delivery of virulence factors to bacterial or eukaryotic cells (Kuehn and Kesty, 2005; Mashburn-Warren and Whiteley, 2006). We have previously shown that OMVs contribute to the delivery of active ClyA cytotoxin, α-haemolysin and CNF1 from *Escherichia coli* to mammalian cells (Wai *et al*., 2003; Balsalobre *et al*., 2006; Kouokam *et al*., 2006). Recently, it was suggested that OMV production is a physiological consequence of Gram-negative bacteria and that OMVs are a component of the matrix of Gram-negative bacterial biofilms (Schooling and Beveridge, 2006). In their study, they found that OMVs from biofilm contained more proteolytic activity than those from planktonic cells. They speculated that OMVs could act as decoys to reduce inimical agents within biofilms before they can attack cells. OMVs are also very promising for different biotechnological applications such as the delivery of antibiotics or as efficient vaccine particles.

In contrast to the extensive research on the biological functions of OMVs, very little is known about the mechanism and regulation of the formation of OMVs. OMV formation has been suggested to be linked to turgor pressure of the cell envelope during bacterial growth (Zhou *et al*., 1998). Release of OMVs is highly dependent on the envelope structure. Defects in proteins either linking the outer membrane to the peptidoglycan layer or involved in a structural network between the inner, outer membranes and the peptidoglycan layer result in the shedding of large amounts of OMVs (McBroom and Kuehn, 2007).

In the past few years, it has become increasingly clear that small non-coding RNAs (sRNAs) regulate many diverse cellular processes, including acid resistance and iron homeostasis (Majdalani *et al*., 2005), and the virulence of pathogens (Romby *et al*., 2006; Toledo-Arana *et al*., 2007). A major class of sRNAs in bacteria functions by base-pairing with target mRNAs, and positively or negatively regulates translation and/or stability of these messages. This class of sRNAs usually requires the RNA chaperone Hfq as a cofactor, which facilitates the interaction between sRNAs and target mRNAs (Storz *et al*., 2004; Valentin-Hansen *et al*., 2004).

Recent systematic searches (Vogel and Sharma, 2005) revealed that *E. coli* expresses close to 100 sRNAs, and the total number of sRNAs in a typical enterobacterium may well range in the hundreds (Hershberg *et al*., 2003; Zhang *et al*., 2004). To date, numerous sRNAs have been predicted in *V. cholerae* (Livny *et al*., 2005), and several of these candidates have been confirmed by Northern blot analysis. Nine sRNAs have been assigned cellular functions in *V. cholerae*: the homologue of *E. coli* RyhB sRNA, which is involved in iron utilization (Davis *et al*., 2005; Mey *et al*., 2005); MicX sRNA, which negatively regulates an uncharacterized outer membrane protein (OMP) and a periplasmic component of a peptide ABC transporter (Davis and Waldor, 2007); seven sRNAs, i.e. Qrr1–Qrr4, CsrB–CsrD, which are involved in quorum-sensing regulation (Lenz *et al*., 2004; 2005).

Here we report on the discovery of a new sRNA in *V. cholerae*, to which we will refer as VrrA (*V**ibrio*regulatory RNA of *omp**A*). VrrA positively regulates OMV release through downregulation of outer membrane protein OmpA. Inactivation of VrrA resulted in increased colonization of *V. cholerae* in the infant mouse colonization assay.

## Results

### Characterization of a new sRNA, VrrA, in *V. cholerae*

**Fig. 1:**
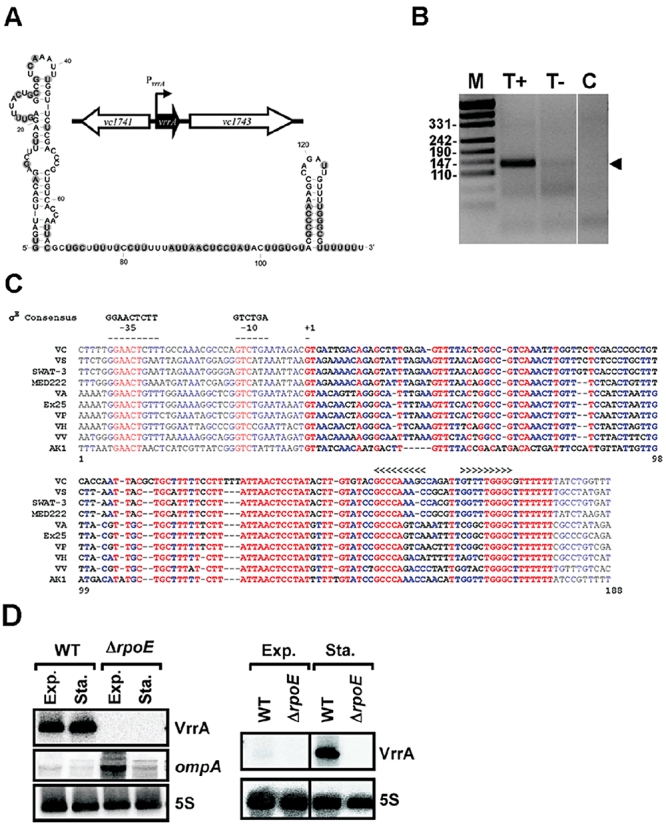
VrrA is conserved among vibrios and *vrrA* promoters contain a σ^E^ consensus motif. A. Secondary-structural prediction (Mfold) for VrrA identified in *V. cholerae.* Grey circles indicate the nucleotides conserved across all VrrAs listed in (C). The insert shows the genomic location of the *V. cholerae vrrA* gene in the *vc1741-vc1743* intergenic region (note that *vc1742* is a very small, 138 bp, predicted open reading frame that has no clear Shine–Dalgarno sequence and only 13 of the 46 codons are overlapping with the *vrrA* locus). B. RACE mapping of 5′ end of *vrrA.* 5′ RACE was carried out as described previously (Urban and Vogel, 2007) to determine the transcription start site (+1) of *vrrA*. Total *V. cholerae* A1552 RNA was linked to a 5′ adaptor RNA without or after treatment with tobacco acid pyrophosphatase (TAP) (lanes T− and T+ respectively). *V. cholerae* A1552 chromosomal DNA served as a control template (lane C). RT-PCR products were separated on a 2% agarose gel. The arrowhead marks the position of the strongly enhanced RT-PCR product upon TAP treatment, which corresponds to the newly initiated VrrA transcript. Cloning of the corresponding bands, followed by sequencing, identified the G residue (marked as +1 in C) as the 5′ end of VrrA RNA. DNA marker sizes (lane M) are given to the left. C. Alignment of *vrrA* genes identified in *V. cholerae* (VC), *V. splendidus* (VS), *V. alginolyticus* (VA), *V. parahaemolyticus* (VP), *V. harveyi* (VH), *V. vulnificus* (VV), *V. shilonii* (AK1), *Vibrionales* bacterium SWAT-3 (SWAT-3), *Vibrio* sp. MED222 (MED222) and *Vibrio* sp. Ex25 (EX25). Annotations for the genes flanking *vrrA* are VC1741/VC1743 for VC, V12B01-03703/03708 for VS, V12G01-19801/19806 for VA, VP1228/VP1229 for VP, VIBHAR-02639/02640 for VH, VV1-2832/2833 for VV, VSAK1-06350/06355 for AK1, VSWAT3-05426/05431 for SWAT-3, MED222-16406/16411 for MED222 and VEx2w-02002168/02002169 for Ex25. The putative σ^E^ binding site is marked as −10 and −35, the transcription start site is labelled as +1, and the terminator is indicated by the arrow heads over the sequence. σ^E^ consensus motif (Vogel and Papenfort, 2006) is shown on top. Numbering of residues follow the *V. cholerae vrrA* sequence. D. Expression of *V. cholerae vrrA* in *Vibrio* (left) and *Salmonella* (right) strains by Northern blot analysis. The *V. cholerae vrrA* gene was cloned in plasmid pTS2 and expressed in *Salmonella* wild type (WT) and isogenic *rpoE* mutant strains. Total RNA was extracted from cultures at exponential phase (Exp.) and stationary phase (Sta.). A 5S rRNA probe was used as loading control. The *Salmonella* strains and 5S rRNA probe were published previously (Papenfort *et al*., 2006).

We became aware of the *vrrA* gene when analysing a mini-Tn*5* transposon mutant (SNW6) from a library of *V. cholerae* El Tor O1 strain A1552 (Vaitkevicius *et al*., 2006), which was found to carry a mini-Tn*5* insertion in the intergenic region between *vc1741* and *vc1743* (Fig. 1A). Inspection and sequence comparison with other *Vibrio* strains of the disrupted region suggested the existence of a previously unrecognized sRNA gene. We successfully validated this prediction by Northern blot analysis, which detected a ∼140 nt RNA expressed from the positive strand in samples of the wild type (Fig. 4) but not of the SNW6 mutant strain (data not shown). Subsequent 5′ RACE analysis of this sRNA species (Fig. 1B) identified the transcription start site (+1) shown in Fig. 1A, which is located approximately 140 bp upstream of a putative Rho-independent terminator downstream. The 5′ RACE analysis was performed to determine the transcription start (+1) site of the *vrrA* downstream gene *vc1743* (Fig. S1). The +1 site of *vc1743* was the same as that of VrrA. This indicates that *vc1743* would be co-transcribed with *vrrA*. However, in the Northern blot analysis, the VrrA probe never detected the reaction band larger than 140 nt. Under the same detection condition, there was no detectable signal with a *vc1743* probe (data not shown). This suggests that, although *vc1743* can be co-transcribed with *vrrA*, the level of readthrough of the proposed terminator (Fig. 1A) is very low under the growth conditions that we used in this study. Interestingly, the *V. cholerae* VrrA promoter region contains a sequence that is a perfect match to the previously reported consensus of promoters recognized by the alternative sigma factor, σ^E^ (Rhodius *et al*., 2006; Skovierova *et al*., 2006). Using blastn searches, we identified *vrrA* homologues in other *Vibrio* species, and all of these genes show conservation of the σ^E^ binding sites in the *vrrA* promoter region (Fig. 1C). In order to analyse the role of RpoE in regulation of *vrrA* expression, we constructed an in-frame deletion *rpoE* mutant and tested the level of *vrrA* expression by Northern blot analysis. The expression of VrrA was totally abolished in the Δ*rpoE* mutant strain (Fig. 1D, left). Furthermore, a cloned copy of *vrrA* with its promoter region (plasmid pTS2) was introduced into *Salmonella typhimurium* strain SL1344 and its otherwise isogenic Δ*rpoE* mutant strain JVS-01028 (Papenfort *et al*., 2006), to test the σ^E^ requirement in the heterologous bacterial system. *S. typhimurium* carrying pTS2 expressed VrrA in a manner that was totally dependent on a functional σ^E^ (Fig. 1D, right). Taken together, our results provided conclusive genetic evidence that *vrrA* expression is directly controlled by the σ^E^ factor.

### OmpA is downregulated by VrrA

**Fig. 2:**
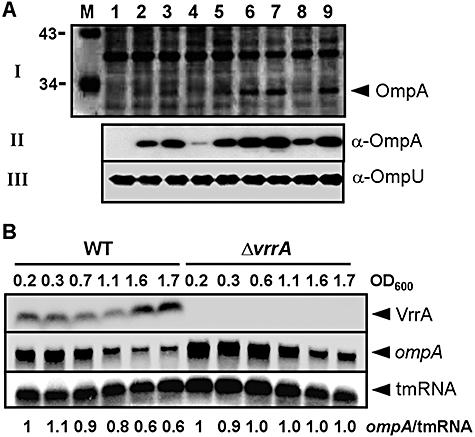
VrrA downregulates *ompA*. A. Coomassie brilliant blue-stained gel (panel I) and Western blot detecting OmpA and OmpU (panel II and III respectively) after SDS-PAGE separation of protein lysates from different derivatives of the *V. cholerae* strain A1552. Lanes 1, DNY10 (Δ*ompA*); 2, A1552 (wild type); 3, DNY7 (Δ*vrrA*); 4, DNY11 (Δ*vrrA*+pvrrA); 5, DNY12 (Δ*vrrA*+vector); 6, DNY8 (Δ*hfq*); 7, DNY9 (Δ*hfq*Δ*vrrA*); 8, DNY16 (Δ*hfq*Δ*vrrA*+pvrrA); 9, DNY17 (Δ*hfq*Δ*vrrA*+vector). Protein marker sizes (lane M) are given to the left in kDa. B. Detection of VrrA and o*mpA* mRNA by Northern blot analysis. Bacterial cells of *V. cholerae* A1552 and the Δ*vrrA* mutant DNY7 were grown in LB and total RNA was isolated at different time points represented by OD_600_ values. The bacteria were in the exponential growth phase between OD_600_ 0.2 and 2.0. We observed no growth rate difference between the wild type and mutant (data not shown). *ompA* and tmRNA levels were quantified and the *ompA*/tmRNA ratio is normalized to the time point of OD_600_ = 0.2.

To investigate the role of VrrA, we made comparisons using the wild-type *V. cholerae* strain A1552 and the *vrrA* deletion strain DNY7. When comparing the whole-cell protein profiles by SDS-PAGE, we noticed that a protein at 34 kDa appeared more abundant in the *vrrA* mutant in comparison with the wild-type strain A1552 (Fig. 2A, panel I; lanes 2 and 3). The protein was identified as the putative outer membrane porin protein OmpA by mass spectrometry analysis. The altered level of OmpA was further confirmed by Western blot analysis using anti-OmpA polyclonal antisera (Fig. 2A, panel II). In order to assess whether the increased level of OmpA in the *vrrA* deletion mutant (DNY7) could be restored by complementation of *vrrA* on a plasmid, we cloned the *vrrA* gene including its promoter region into a low-copy-number plasmid pMMB66HE. The resulting plasmid, pvrrA, as well as a control vector was used to transform strain DNY7, yielding strains DNY11 and DNY12 respectively. Expression of the sRNA from pvrrA was confirmed by Northern blot analysis (Fig. 4). As shown in Fig. 2A, the increased OmpA expression in the *vrrA* mutant carrying the vector plasmid (DNY12) was reduced in the complemented strain DNY11 (Fig. 2A, compare lanes 4 and 5). As a loading control, expression of the outer membrane protein OmpU was measured (Fig. 2A, panel III).

### Absence of *vrrA* increases the level of *ompA* mRNA

Northern blot analyses were performed in order to determine and compare the relative expression levels of VrrA and *ompA* mRNA during growth. Our results showed that *vrrA* was expressed throughout growth and was stable until the stationary phase (Fig. 2B). In contrast, expression of *ompA* was high at the early logarithmic growth phase, but was dramatically reduced when the culture entered the late logarithmic growth phase. In other words, the *ompA* mRNA level decreased upon VrrA accumulation. However, in a strain lacking VrrA, expression of the *ompA* mRNA was maintained at a higher level throughout the exponential growth phase (Fig. 2B). Taken together, these findings strongly suggest a repressive role of VrrA for the expression of the *V. cholerae ompA* gene.

### VrrA represses *ompA* mRNA translation

**Fig. 3:**
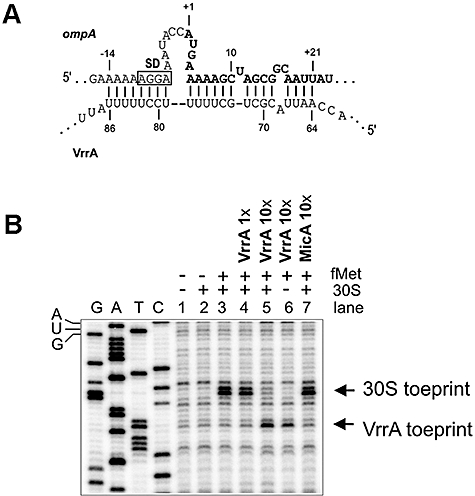
VrrA directly regulates *ompA* mRNA by inhibiting 30S binding. A. Interaction between VrrA and *ompA* mRNA, which was predicted by RNAhybrid program analysis and extended according to the toeprinting analysis (B). B. Toeprinting analysis on *ompA* leader RNA (20 nM). The plus symbol ‘+’ and the minus symbol ‘−’ indicate the presence and absence, respectively, of 30S subunit (20 nM) and fMet initiator tRNA (100 nM). The *ompA* AUG start codon position is shown. Increasing concentrations of VrrA RNA (lanes 4 and 5: 20 and 200 nM) in the reactions inhibit 30S binding, whereas the non-specific control RNA, MicA (lane 7, 200 nM), does not inhibit the toeprint.

Many sRNAs that control OMP synthesis bind to the 5′ untranslated region of the target *omp* mRNAs (Vogel and Papenfort, 2006). Bioinformatic predictions of the VrrA–*ompA* interaction with the RNAhybrid program (Rehmsmeier *et al*., 2004) revealed that a region of VrrA was partially complementary to nucleotides encompassing the ribosome binding site and part of the coding region of the *ompA* mRNA (Fig. 3A). In addition, VrrA homologues from other *Vibrio* species also displayed complementarity to the translation initiation region of the *ompA* mRNA of these strains (Fig. S2). This predicts that VrrA binds to the ribosome binding region of the *ompA* transcript, inhibiting ribosome entry and thus destabilizing this mRNA.

In order to assess the possibility that the regulatory function of VrrA on the *ompA* mRNA was direct, we performed gel-shift and RNA footprint experiments, expecting that VrrA and *ompA* would form a complex *in vitro*. Complex formation was observed and the interaction could be detected upstream and downstream of the ATG in the *ompA* messenger and in the complementary region in VrrA (data not shown).

To obtain direct proof of translational control, we performed toeprinting assays with ribosomal 30S subunits (Hartz *et al*., 1988), testing if VrrA prevented formation of the ternary translation initiation complex (mRNA/30S/tRNA^fMet^) on the *ompA* mRNA (Fig. 3B). An *ompA* mRNA fragment of *V. cholerae*, encompassing the complete 5′ untranslated region (determined by 5′ RACE, Fig. S1) and 75 nt of the coding region, was incubated with purified 30S ribosomal subunit in the presence or absence of uncharged tRNA^fMet^. Subsequently cDNA was synthesized from a primer binding in the *ompA* mRNA coding region. This revealed the typical toeprint signal at position +14/+15 (relative to the AUG start codon of *ompA* mRNA). This signal was lost if the mRNA was incubated with increasing concentrations of VrrA prior to 30S binding (Fig. 3B, lanes 3–5). Instead a new toeprint pattern representing the *vrrA* interaction appeared (Fig. 3B, lanes 5 and 6). *Salmonella* MicA RNA served as a control RNA, and failed to inhibit 30S binding to the *Vibrio ompA* Shine–Dalgarno region (Fig. 3B, lane 7). These experiments show that VrrA specifically and directly pairs with the *ompA* coding region *in vitro* thereby inhibiting ribosome binding.

### VrrA reduces the OmpA level in *hfq* mutant *V. cholerae*

**Fig. 4:**
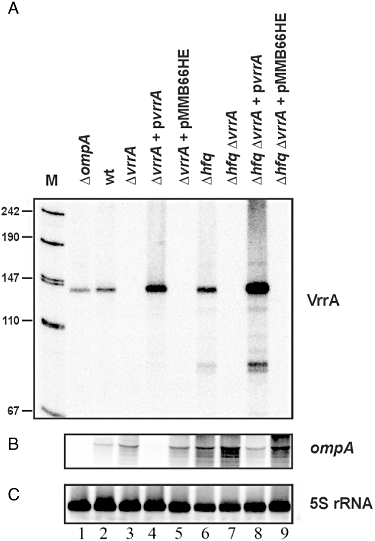
Northern blot analysis of VrrA (A) and *ompA* mRNA (B) levels in *V. cholerae* Δ*vrrA* and Δ*hfq* mutants. Bacterial growth and RNA extraction procedures were as described in *Experimental procedures*. The Northern blot procedure was as described earlier (Papenfort *et al*., 2006). The 5S rRNA (C) was probed as internal control.

To date, many of the sRNAs shown to function by base-pairing to complementary mRNA sequences appear to require involvement of the RNA chaperone protein Hfq (Brennan and Link, 2007). In order to investigate a putative involvement of Hfq for the VrrA-mediated repression of *ompA*, *hfq* mutant derivatives of the wild type and the *vrrA* mutant strain were constructed (DNY8 and DNY9 respectively). In the absence of Hfq, the OmpA level was still elevated by the *vrrA* mutation (Fig. 2A, compare lane 6 with lane 7, panels I and II). Furthermore, OmpA synthesis was still repressed by VrrA expression from plasmid, pvrrA, in a strain lacking Hfq (Fig. 2A, compare lane 8 with lane 9). The Northern blot analysis of *ompA* mRNA showed that the transcript level was threefold higher in the Δ*hfq* Δ*vrrA* double mutant than in the Δ*hfq* single mutant (Fig. 4B, cf. lanes 6 and 7). These data indicated that the VrrA-mediated regulation of OmpA expression did occur in the absence of Hfq. Furthermore, the VrrA overexpression caused a great reduction of the *ompA* mRNA level both in the *hfq* wild-type strain DNY11 and in the *hfq* mutant DNY16 (Fig. 4B, lanes 4 and 8). This suggests strongly that Hfq is not essential for OmpA repression by VrrA although it is also feasible that Hfq can enhance the repression. We also observed that in the *hfq* mutant the basal OmpA protein level was higher (compare lane 2 with lane 6 in Fig. 2A). The apparent repression by Hfq was presumably not strictly dependent on VrrA and could also be mediated by some other sRNA or by a direct interaction of Hfq with the *ompA* transcript as previously proposed for *E. coli* (Vytvytska *et al*., 2000). However, RNA analysis by Northern blot hybridization showed that the total level of VrrA was slightly higher in the Δ*hfq* mutant than in the wild-type strain A1552 which suggests that the Hfq protein somehow might reduce the stability, and thereby the level, of VrrA or indirectly might affect its expression (Fig. 4A, lanes 2 and 6). It has been observed in *V. cholerae* that there is an increased level of *rpoE* expression in the *hfq* mutant (Ding *et al*., 2004). The increased level of RpoE might promote the increased expression of VrrA in *V. cholerae*.

### VrrA promotes OMV production through repressing OmpA synthesis

**Fig. 5:**
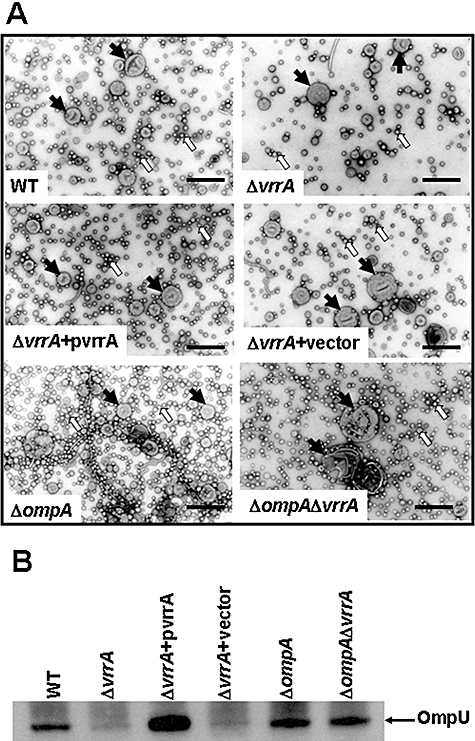
Overexpression of VrrA and deletion of *ompA* lead to increased release of OMVs. A. Electron micrographs of negatively stained samples of OMVs isolated from *V. cholerae* strains: A1552 (WT), DNY7 (Δ*vrrA*), DNY11 (Δ*vrrA*+pvrrA), DNY12 (Δ*vrrA*+vector), DNY10 (Δ*ompA*) and DNY104 (Δ*ompA*Δ*vrrA*). White arrows show small vesicles and black arrows show large vesicles. The bar represents 200 nm in length. B. Immunoblot analysis of the OMV preparations, using anti-OmpU antiserum.

Changes in the outer membrane protein composition in Gram-negative bacteria can result in altered formation and release of OMVs (Sonntag *et al*., 1978). Given that OmpA is an abundant outer membrane protein in *V. cholerae*, we considered that the VrrA regulatory effect on OmpA expression might influence the production of OMVs. To test this hypothesis, we constructed an *ompA* mutant derivative and compared its production of OMVs with the wild-type strain. The result suggested that the lack of OmpA led to more production of OMVs (Fig. 5). The OMVs were visualized by electron microscopy and two different subpopulations of vesicles were observed, i.e. the smaller vesicle with an average diameter of 50 nm indicated by white arrows and larger vesicles with an average diameter of 150 nm, indicated with black arrows (Fig. 5A). The amount of OMVs released was also reflected by the amount of *V. cholerae* major outer membrane protein OmpU (Fig. 5B). As it is a major protein component of the OMVs it was used as a marker in our comparison of OMVs from the different strains. In keeping with the above results, the VrrA-overexpressing strain (DNY11), in which the OmpA level was repressed, produced more OMVs when compared with the Δ*vrrA* vector control strain (DNY12) and the *vrrA* mutant strain alone. In addition, a Δ*ompA* Δ*vrrA* double mutant was constructed to see whether VrrA would have an impact on the OMV production in the absence of OmpA. As shown in Fig. 5A and B, there was no significant difference in the release of OMV when the single Δ*ompA* mutant and double Δ*ompA* Δ*vrrA* mutant were compared. This is consistent with our suggestion that the VrrA effect on OMV release is occurring through OmpA protein regulation.

### VrrA modulates virulence of *V. cholerae*

**Fig. 6:**
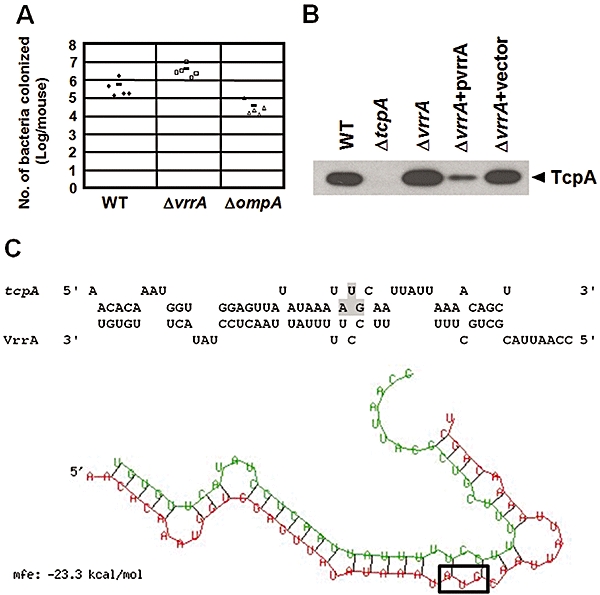
VrrA affects *V. cholerae* virulence through OmpA and TcpA. A. Approximately 10^5^ bacteria of *V. cholerae* wild type and either *vrrA* or *ompA* mutants were inoculated intragastrically into 6-day-old CD-1 (Charles River Laboratories) mice. After a 20 h period of colonization, intestinal homogenates were collected and the bacteria numbers were determined. B. *V. cholerae vrrA* deletion results in increased TCP expression. Western blot detecting TcpA after SDS-PAGE analysis on lysates from different derivatives of the *V. cholerae* strain A1552. A Coomassie brilliant blue-stained gel verifying that there was equal sample loading is shown in Fig. S3. C. Predicted interaction between *tcpA* and VrrA. VrrA single stranded region (nt 61–106) was hybridized with *tcpA* (*vc0828* of *V. cholerae* strain N16961), using RNAhybrid 2.2 online submission (http://bibiserv.techfak.uni-bielefeld.de/rnahybrid/submission.html). Start codon (AUG) of *tcpA* is marked. Sequences of *tcpA* and *vrrA* are in red and green respectively.

It has been shown in *E. coli* that OmpA protein is utilized by the bacteria for adhesion to HeLa epithelial cells and Caco-2 colonic epithelial cells (Torres and Kaper, 2003). *V. cholerae* OmpA shares 47.8% similarity to *E. coli* OmpA. To test whether OmpA has any influence on *V. cholerae* virulence, we used the infant mouse infection model to examine the colonization abilities of *V. cholerae ompA* mutant and wild-type strains. Figure 6A shows that inactivation of *ompA* resulted in a ∼10-fold attenuation in the ability to colonize the infant mouse small intestine. As the *vrrA* mutant overproduced OmpA compared with the wild type (Fig. 2A), we hypothesized that this mutant would behave like the wild type or perhaps even be more virulent in the colonization assay. Indeed, the *vrrA* mutant showed an approximately fivefold increase in colonization ability when compared with the wild-type strain (Fig. 6A). These data suggest that OmpA is important for the colonization ability of *V. cholerae*, and that VrrA RNA may be considered as a regulator that modulates the virulence of *V. cholerae*.

We also attempted to monitor VrrA expression during *V. cholerae* infection of the host by quantitative RT-PCR analysis of RNA from small intestines recovered from the infant mouse infection model (see *Experimental procedures* for details). However, we were not able to detect the VrrA transcript from infected murine small intestinal homogenates, although VrrA was detected in RNA from *in vitro* samples prepared in parallel (data not shown). Although it remains to be verified, we must consider that VrrA is expressed at a rather low level in *V. cholerae* bacteria that are colonizing the host environment.

Earlier studies showed that TCP, a type IV pilus, is required for intestinal colonization (Thelin and Taylor, 1996). The TCP causes aggregation of *V. cholerae* and induces microcolony formation within the intestine. As *vrrA* mutant *V. cholerae* showed increased colonization ability, we were interested to examine whether expression of TCP was influenced by the inactivation of VrrA. We therefore cultivated *V. cholerae* strains in a TCP-inducing growth condition (Iwanaga and Kuyyakanond, 1987) and monitored TCP by Western blot analysis using antisera against the major subunit, TcpA (23 kDa). As shown in Fig. 6B, the TCP level was elevated in the *vrrA* mutant and the effect could be complemented by overexpression of VrrA from a plasmid. Thus, VrrA is either a direct or an indirect regulator of TCP and the increased ability of *vrrA* mutant *V. cholerae* in colonization could at least partially be caused by the increased production of TCP. Our additional analyses suggested that VrrA might interact directly with the *tcpA* mRNA. In the *tcpA* mRNA 5′ region including the translation start and Shine–Dalgarno region there is good sequence complementarity to VrrA as indicated by results from a prediction using the RNAhybrid program (Fig. 6C).

## Discussion

This study describes the discovery of a new *V. cholerae* sRNA (VrrA) that regulates expression of OmpA. VrrA appears to be a direct negative regulator of *ompA* mRNA and unlike other repressors of OmpA synthesis VrrA does not strictly require the RNA chaperone Hfq, suggesting a partly Hfq-independent pathway of *ompA* mRNA repression. We describe a regulatory role of VrrA in the formation and release of OMVs from *V. cholerae* and in modulation of *V. cholerae* virulence. Based on the observation of the σ^E^ consensus binding site, and direct genetic evidence for σ^E^-dependent expression, we propose that VrrA acts as a regulator mediating σ^E^-related stress.

OmpA is a β-barrel protein in the membrane and is highly conserved among Gram-negative bacteria (Delcour, 2002). The biological properties and functions of OmpA have been extensively studied in *E. coli* (Sugawara and Nikaido, 1992; Smith *et al*., 2007). Recently, the *E. coli* MicA and RseX sRNAs, and the *Salmonella* MicA and RybB sRNAs, have been demonstrated to downregulate OmpA levels by a base-paring mechanism, and their functions are Hfq-dependent (Udekwu *et al*., 2005; Douchin *et al*., 2006; Figueroa-Bossi *et al*., 2006; Johansen *et al*., 2006; Papenfort *et al*., 2006; Thompson *et al*., 2007; Udekwu and Wagner, 2007). Importantly, the VrrA of *V. cholerae* is not a homologue of MicA, RybB or RseX, and Hfq is not strictly required for VrrA-mediated downregulation of OmpA. However, it is not ruled out that Hfq can enhance the repression of VrrA on OmpA in a similar fashion to that of the other OmpA repressors, e.g. like MicA in *E. coli* (Udekwu *et al*., 2005). It is noteworthy that a decrease in *ompA* mRNA signals upon entry into late log phase is still observed in the absence of the VrrA RNA (Fig. 2B). We do not yet understand the molecular nature of the additional regulation of *ompA*. The observation that *V. cholerae* OmpA expression was elevated in the *hfq* mutant hints at the existence of additional Hfq-dependent OmpA-regulatory sRNAs in *V. cholerae*. Alternatively, there could be a direct interaction between Hfq and the *ompA* mRNA as been shown to occur in *E. coli* (Vytvytska *et al*., 2000). In addition to VrrA reported here, sRNA RyhB might also have a role in regulating OmpA in *V. cholerae*, although two research groups that characterized RyhB in *V. cholerae* reported opposite conclusions on the regulation of *ompA* by RyhB. Data by Davis *et al*. (2005) revealed that mutation of the *V. cholerae ryhB s*RNA resulted in a 1.7-fold elevation of the *ompA* transcript when the bacteria were grown in minimal medium supplemented with the iron chelator dipyridyl. By comparing *ompA* transcript levels in wild type, *ryhB* mutant and *hfq* mutant *V. cholerae*, the authors concluded that RyhB and Hfq act in conjunction to downregulate expression of the *ompA* gene. However, microarray data by Mey *et al*. (2005) showed that *ompA* was 3.4-fold increased by RyhB. These data imply that OmpA regulation in *V. cholerae* is complex and that the bacteria exploit multiple regulation pathways to fine-tune OmpA expression to adapt to different growth environments.

**Fig. 7:**
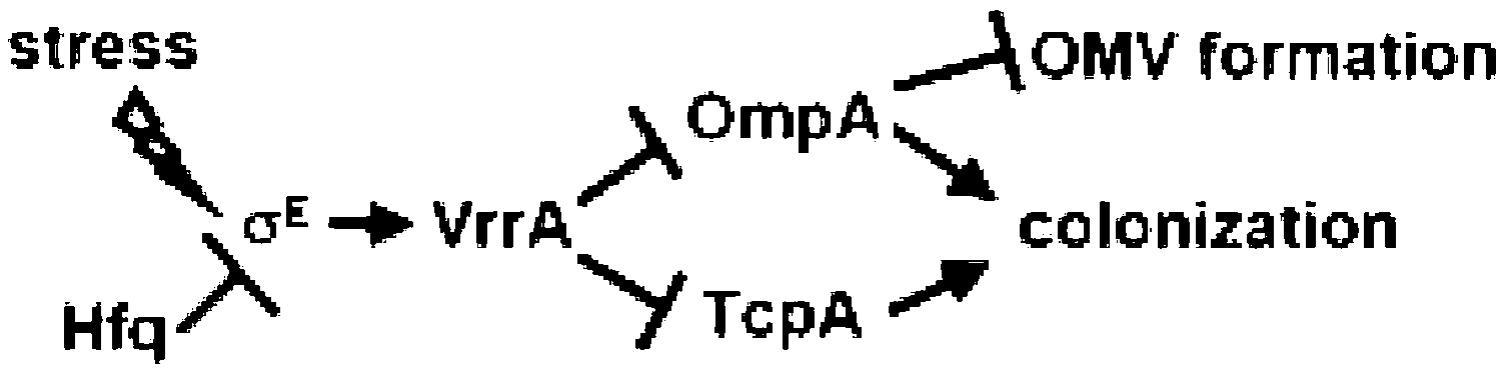
Model of VrrA-mediated OMV release and virulence regulation in *V. cholerae.* See text for the details.

Bacteria respond to changes in their environment by global changes in transcription. These changes in transcription are often accomplished by the induction of alternative sigma factors, which direct RNA polymerase to specific promoters, thereby inducing a set of genes called a regulon to combat the stress. In enteric bacteria one of the key pathways involved in maintaining cell envelope integrity during stress and normal growth is controlled by the alternative sigma factor σ^E^. Previous work established that σ^E^ is essential for viability of *E. coli* (De Las Penas *et al*., 1997) and that it upregulates expression of ∼100 protein-encoding genes that influence nearly every aspect of the cell envelope (Rhodius *et al*., 2006). It has been shown in *E. coli* and *Salmonella* that the MicA and RybB sRNAs are positively and directly controlled by σ^E^ and that these sRNAs collectively act to downregulate all major and many minor OMPs under conditions of membrane stress (Figueroa-Bossi *et al*., 2006; Johansen *et al*., 2006; Papenfort *et al*., 2006; Thompson *et al*., 2007; Udekwu and Wagner, 2007). Based on the observation that the *vrrA* promoter includes a sequence with perfect match to the σ^E^ consensus binding site and there was no *vrrA* expression in the *rpoE* mutants of *V. cholerae* and *S. typhimurium*, it is evident that VrrA acts as a regulator of σ^E^-mediated stress responses. We suggest a model (Fig. 7) that under σ^E^-related stress conditions, typically envelope stress, VrrA is expressed to downregulate the OmpA protein level, which in turn will reduce the envelope stress by producing OMVs. This model is in line with the proposal by McBroom and Kuehn (2007), who suggested that release of OMVs by Gram-negative bacteria is a novel envelope stress response.

It is a remarkable finding that *vrrA* mutant *V. cholerae* showed higher ability in colonizing the infant mouse small intestine. Data presented here would be consistent with the suggestion that the increased colonization ability was due to effects on both OmpA and TCP. We demonstrated that the interaction between VrrA and *ompA* mRNA is direct. Although we do not yet know the exact mechanism underlying VrrA–TCP interaction, there is possibility that VrrA regulates *tcpA* by directly binding to the 5′ untranslated region of *tcpA*, as predicted by RNAhybrid program analysis (Fig. 6C). Considering these findings, we included TCP in our model of VrrA action as summarized in Fig. 7. At the initial stage of *V. cholerae* infection, the σ^E^ level would be low and consequently also VrrA expression would be at a low level. During such a stage, the bacteria would produce OmpA, TCP and other factors that may contribute to colonization of the intestine. When bacterial numbers reach a certain level, or due to the stress from the host, the σ^E^ level elevates, which activates expression of VrrA. After synthesis, VrrA will reduce OmpA and TCP production resulting in reduced interaction with the intestinal mucosa. Concomitantly, the bacteria would produce more OMVs and thereby reduce the envelope stress (Fig. 7).

In summary, we demonstrated that VrrA positively controlled the release of OMVs by negatively controlling the expression of the outer membrane protein OmpA. Moreover, this single small regulatory RNA in *V. cholerae* may influence the bacterial colonization ability as manifested using the mouse intestine model. To the best of our knowledge, this is the first described case of a single *V. cholerae* sRNA that solitarily would affect the virulence of this bacterium. Because VrrA represses rather than promotes virulence in *V. cholerae*, attenuation of colonization ability by some means affecting VrrA expression could be considered as the basis of a strategy for therapeutic intervention in bacterial pathogenicity.

## Experimental procedures

### Bacterial strains and growth conditions

*Vibrio cholerae* strains are derivatives of El Tor Inaba strain A1552 (Yildiz and Schoolnik, 1998). *V. cholerae*, *E. coli* and *S. typhimurium* strains were grown at 37°C in Luria–Bertani (LB) broth supplemented, as appropriate, with carbenicillin at 100 μg ml^−1^ and chloramphenicol at 25 μg ml^−1^. For TCP expression analysis, *V. cholerae* strains were grown at inducing conditions as described previously (Iwanaga and Kuyyakanond, 1987).

### DNA manipulations

In-frame deletions were constructed by the procedures described previously (Vaitkevicius *et al*., 2006). Primer sequences are summarized in Table S1. Deletion of the *vrrA*, *ompA*, *hfq*, *tcpA* and *rpoE* loci in *V. cholerae* strain A1552 resulted in DNY7, DNY10, DNY8, DNY51 and DNY105 respectively. *vrrA* deletion mutant was constructed such that 22 nucleotides upstream from and 91 nucleotides of *vrrA* were removed from the chromosome. Double deletion of *hfq* and *vrrA* in A1552 resulted in DNY9. Double deletion of *vrrA* and *ompA* in A1552 resulted in DNY104. A DNA fragment (304 bp) containing the *vrrA* gene including its putative promoter region was amplified from the A1552 genome and cloned into pMMB66HE (Furste *et al*., 1986) at the HindIII/BamHI sites. The resulting plasmid pvrrA and its vector control (pMMB66HE) were introduced by transformation into DNY7, resulting DNY11 and DNY12; and into DNY8, resulting DNY16 and DNY17 respectively.

In the pvrrA construct, the *vrrA* gene was cloned together with its putative promoter region into the HindIII/BamHI sites in pMMB66HE and consequently the P*tac* promoter in pMMB66HE was located ∼100 bp upstream of *vrrA*'s own promoter. We propose that *vrrA* transcription from pvrrA is driven by its own promoter instead of the P*tac* promoter in pMMB66HE, based on two observations. First, pvrrA was not induced by P*tac* inducers (e.g. IPTG) in the experiments. Second, the size of *vrrA* transcript in strain DNY11 (Δ*vrrA*+pvrrA) was the same as that in the wild-type strain (∼140 nt), and we always observed a single band in the Northern blot analysis (Fig. 4).

The ColE1-based plasmid, pTS2, expressing *Vibrio vrrA* from its own promoter, was constructed based on plasmid pZE12-luc (Lutz and Bujard, 1997). A DNA fragment of pZE12-luc, which lacks the P_Llaco_ promoter region, was amplified by PCR using Phusion-polymerase (Finnzymes) and primers JVO-2512 and pLLacOB, digested with XbaI, resulting the backbone for pTS2. The *V. cholerae vrrA* gene was PCR-amplified using primers JVO-2639 and JVO-2640. JVO-2639 binds 100 nt upstream of the +1 site of *vrrA* and carries a 5′ monophosphate for cloning; JVO-2640 binds 80 nt downstream of the *vrrA* terminator and will add an XbaI site to the PCR product. Following XbaI digestion, the product was ligated to the backbone, to yield plasmid pTS2 upon transformation.

### RNA preparation and Northern blot analysis

RNA was prepared using Trizol according to the manufacturer's instructions (Invitrogen) and quantified on a NanoDrop ND-1000 Spectrophotometer (NanoDrop Technologies). For Northern blotting, 20 μg of total RNA was separated on a formaldehyde:agarose gel prior to blotting as previously described (Sheehan *et al*., 1995). The Hybond-N membrane (GE Healthcare) was subsequently hybridized with ^32^P-labelled gene-specific probes (Table S1). Northern blots were exposed to a phosphorimager screen and scanned on a Storm^TM^ phosphorimager (Molecular Dynamics, USA). Quantification was performed using ImageQuant^TM^ software (Molecular Dynamics).

### 5′ RACE analysis

5′ RACE was carried out as described previously (Urban and Vogel, 2007) to determine the transcription start sites of *vrrA*, *ompA* and *vc1743*. Total RNA obtained on strain *V. cholerae* A1552 was used for cDNA generation. Oligo TY2, VC2213-rev and TIS-31 (Table S1) were used as *vrrA*-, *ompA*- or *vc1743*-specific primers in PCR. PCR products were separated on a 2% agarose gel (Fig. 1B for *vrrA* and Fig. S1 for *ompA* and *vc1743*), gel-eluted and used as template for sequencing.

### Toeprinting analysis

Toeprinting reactions were carried out as described (Sharma *et al*., 2007) with few modifications. An unlabelled *ompA* mRNA fragment (0.2 pmol; 176 nt; T7 template amplified with primers JVO-2784/-2871), and 0.5 pmol of 5′-end-labelled primer JVO-2871 complementary to the *ompA* coding region were annealed. For inhibition analysis, 0.2 and 2 pmol of VrrA RNA (134 nt, T7 template amplified with JVO-2782/-2783) or 2 pmol of control RNA (*Salmonella* MicA) were added. See the figure legend of Fig. 3 for final concentrations of other components.

### Isolation of OMVs

Outer membrane vesicles were isolated from culture supernatants as previously described (Wai *et al*., 2003).

### SDS-PAGE and Western blot analysis

Protein samples were prepared from equal amount of bacteria cells after grown overnight unless otherwise indicated. The standard SDS-PAGE procedure was used (Laemmli, 1970). Gels were stained with Coomassie brilliant blue. Western blot analyses were performed as described earlier (Vaitkevicius *et al*., 2006), using polyclonal anti-OmpA, anti-OmpU and anti-TcpA antisera.

### Electron microscopy

Procedures for electron microscopy were essentially as described earlier (Wai *et al*., 2003).

### Infant mouse competition assay

Approximately 10^5^ wild type and either *vrrA* or *ompA* mutants were inoculated intragastrically into 6-day-old CD-1 (Charles River Laboratories) mice. Mice were sacrificed after 20 h and bacteria colonizing the intestines were quantified as described previously (Gardel and Mekalanos, 1996).

### Analysis of VrrA expression in the suckling mouse intestine

For analysis of gene expression in the suckling mouse intestine, mice were infected with A1552 as described (Camilli and Mekalanos, 1995) with an inoculum of around 10^5^ colony-forming units (cfu) in 50 μl of LB broth. RNA was isolated from three (first experiment) or six (second experiment) separate small intestines of infected CD-1 mice 24 h post infection. RNA was isolated in parallel from an equal number of separate *in vitro* OD_600_ = 0.5 LB-broth cultures. RNA extraction, removal of chromosomal DNA contamination, random-primed reverse transcription and qPCR were carried out as described previously (Schild *et al*., 2007).

### Mass spectrometry peptide sequencing

Proteins of interest were cut out from gel and analysed at Alphalyse (Denmark) for mass spectrometry.
