# Supplementary material for: A new Vibrio cholerae sRNA modulates colonization and affects release of outer membrane vesicles
Source: Mol Microbiol. 2008 Aug 15;70(1):100–11. doi: 10.1111/j.1365-2958.2008.06392.x (PMC2628432; doi:10.1111/j.1365-2958.2008.06392.x)
Supplement: Supplementary file 1 [file mmi0070-0100-SD1.pdf]

Figure S1

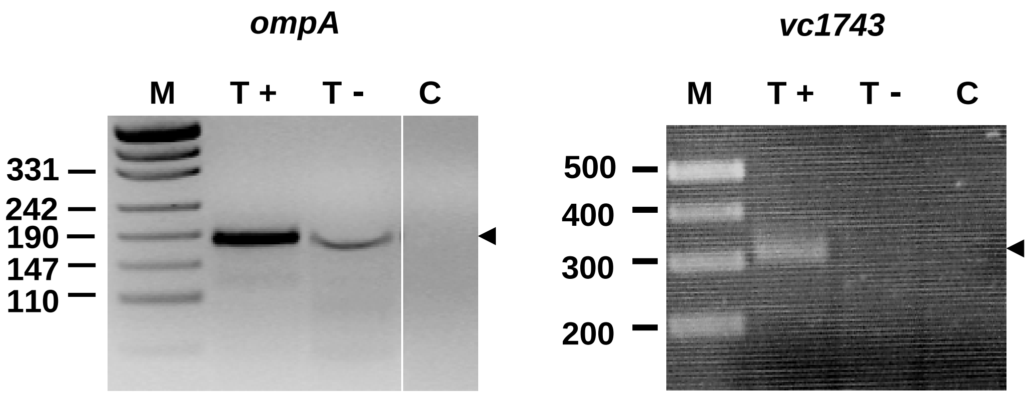

Figure S2

V. cholerae

```
ompA  5′-A          AAUACCAUGA       U    GCAAUUAUUU      AC    A  -3′
            AAAAAGGA          AAAAGC AGCG           CAGCG  GUU
            UUUUUCCU          UUUUCG UCGC           GUCGC  CAG
sRNA  3′-A                              AUUAACCACU      C     C  -5′
```

V. parahemolyticus

```
ompA  5′-G     A  C     U       C                 U -3′
          AGGA U  AAUAA GAAAAAA UAGCAGC  GGUAAUU
          UCCU A  UUAUU CUUUUUU GUCGUUG  UUGUUAA
sRNA  3′-A     C  A             C       CA        U -5′
```

V. harveyi

```
ompA  5′- G     A  C     U      A U     C   A U -3′
           AGGA U  AAUAA GAAAAA C AGCAG GUA U
           UCCU A  UUAUU CUUUUU G UCGUU CAU G
sRNA  3′- A     C  A            C       A   C U -5′
```

V. splendidus

```
ompA  5′-C            AAAGA    ACU   CA   UUCUU   G  U -3′
          GUAAGGAAAA       UGCA   UAA   AAG     AAA GC
          UAUUCCUUUU       ACGU   AUU   UUC     UUU CG
sRNA  3′-U                     C     AA              U -5′
```

V. alginolyticus

```
ompA  5′-G     A C     U       U         G    U -3′
          AGGA U AAUAA GAAAAAA UAGCAGCG UAAU
          UCCU A UUAUU CUUUUUU GUCGUUGC AUUG
sRNA  3′-A     C A             C            U -5′
```

V. vulnificus

```
ompA  5′-A      A      U U   A   AU    C   C -3′
           AGGA   AAUA A GA  AAA  UAGC GU
           UCCU   UUAU U CU  UUU  GUCG UG
sRNA  3′-A    CAA            A    C    U   C -5′
```

Vibrio sp. Ex25

```
ompA  5′-G    A C       U       U        G    U -3′
          AGGA U  AAUAA GAAAAAA UAGCAGCG UAAU
          UCCU A  UUAUU CUUUUUU GUCGUUGC AUUG
sRNA  3′-A    C A               C             U -5′
```

Vibrionales SWAT3

```
ompA 5′- U    A        UAUC            ACGC    C
           UUAA AAGGAAA     AUG    AAU     UAA   GGC -3′
           AAUU UUCCUUU     UAC    UUA     AUU   UCG -5′
sRNA 3′- C    A                GUCA           CUU
```

Vibrio sp. MED222

```
ompA 5′- U    A        UAUC            ACGC    C
           UUAA AAGGAAA     AUG    AAU     UAA   GGC -3′
           AAUU UUCCUUU     UAC    UUA     AUU   UUG -5′
sRNA 3′- C    A                GUCA           CUU
```

V. shilonii

```
                         AAUAC    UUC       C
ompA 5′- CAGAAAAAAGGA         AAUG    GAAAAA CAGU UGU -3′
sRNA 3′- GUUUUUUAUCCU         UUAU    CUUUUU GUCG AUA -5′
                          CAA       U       C    U
```

Figure S3

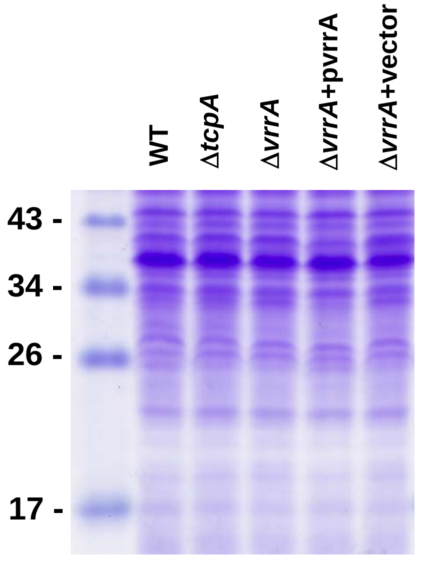

Supplementary Figure legends

Fig. S1. RACE mapping of 5´ ends of *ompA* and *vc1743*. 5´ RACE was carried out as described previously (Urban & Vogel, 2007) to determine the transcription start sites (+1) of *ompA* and *vc1743*. Total *V. cholerae* A1552 RNA was linked to a 5' adaptor RNA without or after treatment with tobacco acid pyrophosphatase (TAP) (lanes T- and T+, respectively). *V. cholerae* A1552 chromosomal DNA served as a control template (Lane C). RT-PCR products were separated on a 2% agarose gel. The arrowhead marks the position of the strongly enhanced RT-PCR product upon TAP treatment, which corresponds to the newly initiated transcripts. Cloning of the corresponding bands, followed by sequencing, identified the G residue at -101 nt upstream from AUG of *ompA* mRNA as the 5´end of *ompA* transcript. The +1 site of *vc1743* was the same as that of *vrrA*. DNA marker sizes (lane M) are given to the left.

Fig. S2. Predicted interaction between *ompA* and VrrA homologs in different *Vibrio* species. *ompA* and *ompA* homologous sequences were aligned with VrrA and VrrA homologous sequences from different *Vibrio* species, respectively. *ompA* sequences were: *V. cholerae*, VC2213; *V. parahemolyticus*, VP0764; *V. harveyi*, VIBHAR_01273; *V. alginolyticus*, V12G01_15380; *V. splendidu*s, V12B01_07036; *V. vulnificus*, VV2_0559; *Vibrio* sp. EX25, VEx2w_02002870; *Vibrionales* SWAT3, VSWAT3_14627; *Vibrio* sp. MED222, MED222_20829; *V. shilonii*, VSAK1_10973. VrrA sequences were identical to those in Fig. 1A

Fig. S3. Coomassie brilliant blue-stained SDS-PAGE gel of bacterial cell lysates from *V. cholerae* wild type strain A1552 and its mutant derivatives. Protein marker sizes (lane M) are given to the left in kDa.

Table S1. Oligonucleotides used in this study

Primer	5’ to 3’ sequence	Restriction site
uraD1	CGCTCTAGAACAAGGTTTCGCGAGTCAAG	XbaI
uraD2	CCCATCCACTAAACTTAAACAGCAAAGAGTTCCAAAAGTTCG	
uraD3	TGTTTAAGTTTAGTGGATGGGCTCCTATACTTGTGTACGCC	
uraD4	CGCTCTAGATCAACGTAAAACTCCAAGCG	XbaI
ompAD1	CGCTCTAGATAAGGTTTTACATCGCTCAG	XbaI
ompAD2	CCCATCCACTAAACTTAAACAACTGAATAATTTACGGTAATC	
ompAD3	TGTTTAAGTTTAGTGGATGGGTTTCATGGTATTTCCTTTTTTC	
ompAD4	CGCTCTAGAAAGCACTGCATCACGTGATC	XbaI
hfqD1	GCGTCTAGAACATTATATGCCAGAGACGATC	XbaI
hfqD2	CCCATCCACTAAACGGTACCATAGAGATTGCCCCTTAGCCAT	
hfqD3	TGGTACCGTTTAGTGGATGGGTCTGAAGAGTAATTCTTTGCA	
hfqD4	GCGTCTAGAATGTCTTTCTAAGTGCGTCCAG	XbaI
tcpAD1	CGCTCTAGAACAGTCAAAGTGACTGAAAG	XbaI
tcpAD2	CCCATCCACTAAACTTAAACATTGCATATTTATATAACTCCAC	
tcpAD3	TGTTTAAGTTTAGTGGATGGGAGTTAATCTACACCATTATC	
tcpAD4	CGCTCTAGAAAGGATGTACATAGGTTGAG	XbaI
rpoED1	CGCTCTAGACATCCAACTCGACATTTC	XbaI
rpoED2	CCCATCCACTAAACTTAAACAGTTCATTCGAGCGGTCAC	
rpoED3	TGTTTAAGTTTAGTGGATGGGCTTCTGTAACGCAAATTCC	
rpoED4	CGCTCTAGATCATTTTCAACGCCTGAG	XbaI
TY1	GGAACTCTTTGCCAAACGCC	
TY2	GGGCGTACACAAGTATAGGAG	
TY5	CCCAAGCTTCATTTTCTCGCCATGGTCTG	HindIII
TY6	CCCGGATCCGCCAATGAACCGACTTGAAC	BamHI
OMA1	AATTATTTCAGCGACGTTAC	
OMA2	ACAACCTGATCATCATCTTC	
tmRNA-F	TCGCAAACGACGAAAACTACG	
tmRNA-R	TAGATCTCGCGCTTCATCCC	
vrrAqF	CCAAACGCCCAGTCTGAATAGA	
vrrAqR	GGGCGTACACAAGTATAGGAGT	
Vspp-16SF-156	CGTAAAGCGCATGCAGGTG	
Vspp-16SR-157	CTTCGCCACCGGTATTCCTT	
JVO-2782	GTTTTTTTTAATACGACTCACTATAGGTGATTGACAGAGCTTTGAG	
JVO-2783	ACGCCCAAAACAATCT	

JVO-2784	GTTTTTTTTAATACGACTCACTATAGGACAAAAAGGTGATCT GGC	
JVO-2871	GACATACACCTCTGCCACTGC	
JVO-367	ACTGACATGGAGGAGGGA	BseRI
VC2213-R	GTTTTTGCTAGCAGAAGCAAAAAGTAACG	NheI
TIS-31	GTTTTTGCTAGCATCAAACTCTCTCCGAGTGAC	NheI
JVO-2512	tttttaagcttTAATAATGGTTTCTTAGACGT	
pLLacOB	CGCACTGACCGAATTCATTAA	
JVO-2639	P-GCTAAGTGCTTGCAGCAC	
JVO-2640	gttttTCTAGACCACTTATCGCCGTGCTC	

The restriction sites are underlined; primers uraD1-4, ompAD1-4, hfqD1-4, tcpAD1-4 and rpoED1-4 were used for mutant constructions of *vrrA*, *ompA*, *hfq*, *tcpA* and *rpoE*, respectively. Primers TY1/TY2, OMA1/OMA2 and tmRNA-F/tmRNA-R were used for PCR to generate probes against *vrrA*, *ompA* and *tmRNA*, respectively, in Northern Blot analysis. vrrAqF/vrrAqR (within the TY1/TY2 region) designed using PrimerQuest (Integrated DNA Technologies) or Vspp-16SF-156/Vspp-16SR-157 primer pairs (Gonzalez-Escalona *et al.*, 2006) were used to generate qRT-PCR products for *vrrA* or the 16S rRNA control respectively. Primers TY5 and TY6 were used for cloning of *vvrA* into pMMB66HE at *Hind*III/*Bam*HI site. JVO-2782/-2783/-2784/-2871 were used in the toeprinting analysis. JVO-367 (Urban & Vogel, 2007) in combination with TY2, VC2213-R or TIS-31 were used in the 5´ RACE experiments for *vrrA*, *ompA* and *vc1743*, respectively. Oligos JVO-2512 (Urban & Vogel, 2007), pLLacOB (Urban & Vogel, 2007), JVO-2639 and JVO-2640 were used to construct pTS2.

References

Gonzalez-Escalona, N., A. Fey, M. G. Hofle, R. T. Espejo & A. G. C. (2006) Quantitative reverse transcription polymerase chain reaction analysis of *Vibrio cholerae* cells entering the viable but non-culturable state and starvation in response to cold shock. *Environ Microbiol* **8:** 658-666.

Urban, J. H. & J. Vogel. (2007) Translational control and target recognition by *Escherichia coli* small RNAs *in vivo*. *Nucleic Acids Res.* **35:** 1018-1037.
